# Preoperative intravenous iron to treat anaemia before major abdominal surgery (PREVENTT): a randomised, double-blind, controlled trial

**DOI:** 10.1016/S0140-6736(20)31539-7

**Published:** 2020-10-24

**Authors:** Toby Richards, Ravishankar Rao Baikady, Ben Clevenger, Anna Butcher, Sandy Abeysiri, Marisa Chau, Iain C Macdougall, Gavin Murphy, Rebecca Swinson, Tim Collier, Laura Van Dyck, John Browne, Andrew Bradbury, Matthew Dodd, Richard Evans, David Brealey, Stefan D Anker, Andrew Klein

**Affiliations:** a Division of Surgery, University of Western Australia, Fiona Stanley Hospital, Perth, WA, Australia; b Institute of Clinical Trials and Methodology, University College London, London, UK; c Division of Surgery, University College London, London, UK; d Royal Marsden NHS Foundation Trust, London, UK; e Department of Anaesthesia, Royal National Orthopaedic Hospital, London, UK; f Department of Renal Medicine, King's College Hospital, London, UK; g NIHR Leicester Biomedical Research Centre, Department of Cardiovascular Sciences, University of Leicester, Leicester, UK; h Clinical Trials Unit, London School of Hygiene & Tropical Medicine, London, UK; i School of Public Health, University College Cork, Cork, Ireland; j University Department of Vascular Surgery, Birmingham University, Solihull Hospital, Solihull, UK; k Department of Cardiology (CVK), Berlin Institute of Health Center for Regenerative Therapies, and German Centre for Cardiovascular Research partner site Berlin, Charité Universitätsmedizin Berlin, Berlin, Germany; l Department of Anaesthesia and Intensive Care, Royal Papworth Hospital, Cambridge, UK

## Abstract

**Background:**

Preoperative anaemia affects a high proportion of patients undergoing major elective surgery and is associated with poor outcomes. We aimed to test the hypothesis that intravenous iron given to anaemic patients before major open elective abdominal surgery would correct anaemia, reduce the need for blood transfusions, and improve patient outcomes.

**Methods:**

In a double-blind, parallel-group randomised trial, we recruited adult participants identified with anaemia at preoperative hospital visits before elective major open abdominal surgery at 46 UK tertiary care centres. Anaemia was defined as haemoglobin less than 130 g/L for men and 120 g/L for women. We randomly allocated participants (1:1) via a secure web-based service to receive intravenous iron or placebo 10–42 days before surgery. Intravenous iron was administered as a single 1000 mg dose of ferric carboxymaltose in 100 mL normal saline, and placebo was 100 mL normal saline, both given as an infusion over 15 min. Unblinded study personnel prepared and administered the study drug; participants and other clinical and research staff were blinded to treatment allocation. Coprimary endpoints were risk of the composite outcome of blood transfusion or death, and number of blood transfusions from randomisation to 30 days postoperatively. The primary analysis included all randomly assigned patients with data available for the primary endpoints; safety analysis included all randomly assigned patients according to the treatment received. This study is registered, ISRCTN67322816, and is closed to new participants.

**Findings:**

Of 487 participants randomly assigned to placebo (n=243) or intravenous iron (n=244) between Jan 6, 2014, and Sept 28, 2018, complete data for the primary endpoints were available for 474 (97%) individuals. Death or blood transfusion occurred in 67 (28%) of the 237 patients in the placebo group and 69 (29%) of the 237 patients in the intravenous iron group (risk ratio 1·03, 95% CI 0·78–1·37; p=0·84). There were 111 blood transfusions in the placebo group and 105 in the intravenous iron group (rate ratio 0·98, 95% CI 0·68–1·43; p=0·93). There were no significant differences between the two groups for any of the prespecified safety endpoints.

**Interpretation:**

Preoperative intravenous iron was not superior to placebo to reduce need for blood transfusion when administered to patients with anaemia 10–42 days before elective major abdominal surgery.

**Funding:**

UK National Institute of Health Research Health Technology Assessment Program.

## Introduction

Preoperative anaemia affects 30–60% of all patients undergoing major elective surgery and is associated with an increased risk of blood transfusion, in-hospital complications, delayed hospital discharge, and poor recovery.^1^, ^2^ The commonest cause of anaemia is iron deficiency, either due to nutritional deficiency or blood loss leading to a state of absolute iron deficiency characterised by low iron stores. Surgical patients often have inflammation or chronic diseases that cause disruptions to the normal pathways for iron transport and iron metabolism. Specifically, the master regulator of iron metabolism, hepcidin, is elevated, which inhibits iron transport out of cells. This process prevents dietary iron absorption and promotes sequestering of available iron into macrophages, leading to a state of functional iron deficiency that in turn leads to anaemia of chronic disease.^3^, ^4^, ^5^ Consequently, treatment of anaemic surgical patients with oral iron is considered ineffective.^6^

In contrast, the use of intravenous iron bypasses these hepcidin-mediated pathways and can result in improvements in haemoglobin concentration, functional performance, and quality of life in patients with anaemia of chronic disease seen with kidney failure,^7^ heart failure,^8^ inflammatory bowel disease,^9^ and women's health.^10^, ^11^

International treatment guidelines recommend that patients undergoing surgery with an expected blood loss of 500 mL or more should be screened for anaemia at least 2 weeks before surgery, with a recommendation that anaemia should be treated with intravenous iron.^12^, ^13^ However, the diagnosis of iron deficiency in patients with preoperative anaemia is not clear and the use of intravenous iron in patients before surgery to correct anaemia and reduce blood transfusion is based on very low-quality evidence.^14^ To address this knowledge gap, we conducted a double-blind, placebo-controlled, randomised trial to compare the clinical effectiveness of intravenous iron therapy given to patients with anaemia 10–42 days before major open elective abdominal surgery. We hypothesised that intravenous iron would be superior to placebo with respect to patient outcomes of blood transfusion, death, adverse events, and quality of life.

**Research in context**

**Evidence before this study**

Preoperative anaemia is common in surgical patients and associated with worse patient outcomes including increased need for blood transfusion, postoperative complications, and longer hospital stay. The commonest cause is iron deficiency due to blood loss from the underlying disease for which the patient is having surgery (eg, gastrointestinal cancer) or indirectly due to inflammation from the disease process or secondary to patient comorbidities that disrupt iron absorption and iron transport leading to anaemia of chronic disease. In the preoperative setting, oral iron has a limited role as the absorption is blocked and there is little time before surgery to replenish iron stores. Intravenous iron has been proposed as an alternative owing to its ability to bypass normal iron transport pathways and deliver a large dose of iron directly to the bone marrow to treat anaemia.

The National Health Service (NHS) England Commissioning for Quality and Innovation scheme for 2020–21 recommends that patients undergoing surgery with an expected blood loss of 500 mL or more should be screened for anaemia at least 2 weeks before surgery and treated with iron therapy if necessary. The guidance claims that “Improved compliance would reduce blood transfusion rate for major blood loss surgeries, reducing the occurrence of patient safety risks associated with blood transfusion including fluid overload, infection and incorrect blood transfusions being given.” However, this is based on guidance from the UK's National Institute for Health and Care Excellence (NICE) that reported only very low quality of evidence.

A Cochrane review of iron therapy for preoperative anaemia was updated in December, 2019. This review concluded that the use of iron therapy for preoperative anaemia does not show a clinically significant reduction in allogeneic blood transfusion compared with no iron therapy but that further, well designed, adequately powered, randomised controlled trials were required to determine the true effectiveness of iron therapy for preoperative anaemia.

**Added value of this study**

The primary results of our trial show no evidence of clinical benefit in giving intravenous iron preoperatively to patients undergoing major abdominal surgery and provide the highest quality of evidence to date, with sufficient statistical power to make strong inferences about effectiveness.

**Implications of all the available evidence**

The evidence base now suggests that current guidance on preoperative iron therapy by, for example, NHS England and NICE, should be revised and now state that preoperative iron therapy is not recommended in major elective surgery patients with anaemia.

## Methods

### Study design and participants

Preoperative intravenous iron to treat anaemia in major surgery (PREVENTT) was a multicentre, double-blind, parallel-group, randomised study in adult patients identified with anaemia 10–42 days before major open abdominal surgery at 46 UK tertiary care centres. The original study protocol is available online and methodological details of the trial are presented in the appendix and described in brief here. The trial was approved by the UK National Research Ethics Committee, East of England.

Eligible participants, identified in preoperative hospital visits, were older than 18 years of age and had haemoglobin less than 130 g/L for men and 120 g/L for women. Specific iron studies were not part of the primary inclusion criteria but formed part of the predefined subgroup analysis. Major surgery was defined as surgery lasting more than 1 h with an operative code of major, major plus, or complex major operation. Exclusions included laparoscopic surgery, concurrent infection, bodyweight of less than 50 kg, known chronic liver disease, another cause for anaemia (eg, haemoglobinopathy) or acquired iron overload, known family history of haemochromatosis or thalassaemia, or transferrin saturation greater than 50%. Full eligibility criteria is included in the appendix (p 6). Participants provided written informed consent.

### Randomisation and masking

Randomisation was done by trained staff members using a secure web-based service through the Clinical Trials Unit at the London School of Hygiene & Tropical Medicine. The web-based service was provided by an independent research support organisation. Randomisation was 1:1, with allocation concealment that used minimisation, considering baseline haemoglobin (<100 *vs* ≥100 g/L), age (<70 *vs* ≥70 years), centre, and operation type (major, major plus, complex major). Because the intravenous iron was a dark-brown solution that is easily distinguishable from the saline placebo, dedicated unblinded study personnel were responsible for the preparation and administration of the study drug but had no other involvement in the trial. To ensure blinding of the participants, their skin was swabbed with iodine, and the study treatment was shielded from vision (light protection bags) and infused through black tubing. Other clinical and research staff were blinded to the treatment allocated.

### Procedures

Intravenous iron was administered as a single 1000 mg dose of ferric carboxymaltose (Ferinject, Vifor Pharma Management, Zurich, Switzerland) in 100 mL normal saline, and placebo was 100 mL normal saline, both given as an infusion over 15 min. Participants were monitored for adverse events or signs of hypersensitivity during and for at least 30 min after treatment. There was no other change to the patient's normal surgical pathway. Clinical assessments and patient-reported outcomes were recorded at enrolment, during the index hospital admission, and 8 weeks and 6 months after the index surgery.

### Outcomes

The trial had two coprimary outcomes: risk of the composite endpoint of blood transfusion or death and the number of blood transfusion episodes from randomisation until 30 days after the index operation. A blood transfusion was defined as receiving 1 unit (or part thereof) of packed red blood cells or any other blood component. A blood transfusion episode referred to the administration of 1 or more units of packed red blood cells or any other blood components in one 24-h period and a large transfusion where 4 or more blood transfusions were given in one episode.

Secondary endpoints included: total number of units of packed red blood cells or blood components transfused (excluding large blood transfusions) at 30 days and 6 months after surgery, change in haemoglobin concentration from randomisation to day of the index operation (before surgery) and at 8 weeks and 6 months after surgery, postoperative complications, intensive care unit (ICU) and total hospital length of stay, days alive and out of the hospital from the date of the planned surgery until 30 days after the index operation, readmission to the hospital at 8 weeks and 6 months postoperatively, and health-related quality of life (HRQoL). Haemoglobin at baseline (pre-randomised treatment) and on the day of the index operation (before surgery) were measured by a central laboratory; all other measurements were from local laboratories. Local sites were responsible for data collection. De-identified data were adjudicated centrally before data lock and unblinding for analysis. HRQoL was measured by the Multidimensional Fatigue Inventory (MFI) questionnaire^15^ and the European Quality of Life: 5 Dimensions 5 Levels (EQ-5D-5L)^16^ score on the day of the index operation (before surgery) and 8 weeks and 6 months after the index operation (appendix pp 7–8).

Prespecified safety endpoints were serious adverse events, suspected unexpected serious adverse events, adverse reactions to trial therapy, and development of perioperative acute kidney injury. All serious adverse events were reviewed by one of the research fellows (BC, AB, or SA) and then adjudicated and coded by LVD using standard Medical Dictionary of Regulatory Authorities (MedDRA). The MedDRA codes were then checked by the research fellows. All reviews were carried out blinded to the treatment.

### Statistical analysis

Assuming an anticipated blood transfusion risk of 40% in the placebo group, we calculated that 500 patients would provide 90% power at the 5% significance level to detect an absolute reduction of 14% for the composite coprimary endpoint of blood transfusion or death by 30 days after the surgery, allowing for 5% loss to follow-up.

The primary analysis was by intention to treat, including all randomly assigned patients with data available for the primary endpoints; safety analysis included all randomly assigned patients according to the treatment received. For the first coprimary endpoint, a risk ratio and 95% CI were calculated using binomial regression, and a p value was calculated using a likelihood ratio test. For the second coprimary endpoint, a rate ratio and 95% CI were calculated using a negative binomial regression model, and a likelihood ratio test p value was calculated. Because some patients died before the end of the study, the length of each patient's period of observation was included as an exposure in the model. To account for multiple testing, a Benjamini–Hochberg procedure with a 5% false discovery rate was used to determine statistical significance for the coprimary endpoints.^17^ The analysis of the coprimary endpoints was repeated for the per-protocol population, excluding patients who did not have the trial treatment or undergo surgery, had their operation outside the prescribed timelines, had an operation not classified as major open abdominal surgery, or withdrew consent. The analysis of the two coprimary endpoints was repeated, adjusting for variables included in the stratification for randomisation (age, baseline haemoglobin, and planned operation type) and from baseline to 6 months after the index operation as secondary endpoints.

Haemoglobin concentration was analysed using analysis of covariance (ANCOVA), adjusting for baseline haemoglobin (appendix p 7). Total number of units of blood or blood components (excluding large blood transfusions) and number of hospital readmissions for complications (including repeat readmissions) were analysed using negative binomial regression. ICU length of stay, total hospital length of stay, and days alive and out of the hospital were analysed using linear regression. HRQoL outcomes (MFI and EQ-5D-5L) were analysed using ANCOVA adjusting for baseline measurements. All-cause mortality, postoperative complications, and readmission to hospital for complications were analysed using the same method as for the first coprimary endpoint. Predefined subgroup analyses for the coprimary endpoints were done for age (<70 *vs* ≥70 years), sex (male *vs* female), body-mass index (<30 *vs* ≥30 kg/m^2^), operation type (major, major plus, or complex major), haemoglobin concentration (<100 *vs* ≥100 g/L), ferritin concentration (<100 *vs* ≥100 ng/mL), and transferrin saturation (<20% *vs* ≥20%). Subgroup analyses were considered supplementary and were not adjusted for multiple testing. More details of the statistical methods are provided in the clinical trial protocol and the statistical analysis plan. All analyses were done with Stata software version 15.0. The trial was registered, ISRCTN67322816.

### Role of the funding source

The study sponsors had no role in the study design, the collection, analysis, and interpretation of data, the writing of the report or in the decision to submit the paper for publication. The corresponding author had full access to all the data in the study and had final responsibility for the decision to submit for publication.

## Results

**Figure 1:**
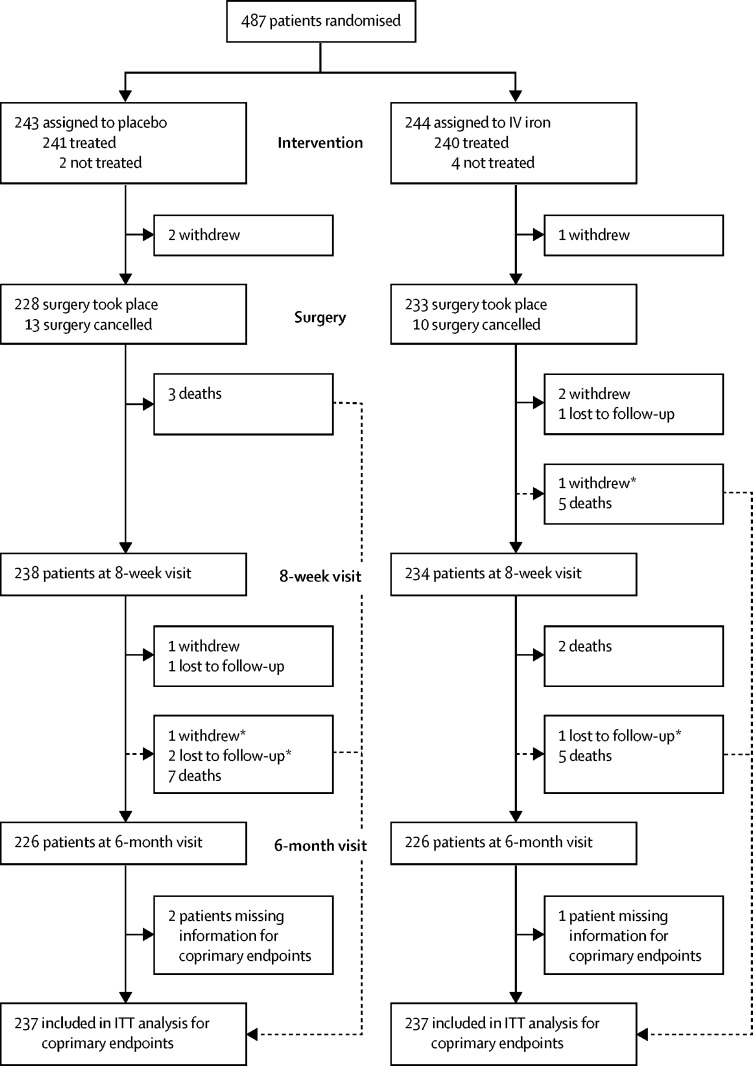
Trial profile ITT=intention-to-treat. IV=intravenous. *Patient had blood transfusion before withdrawal or loss to follow-up and is therefore included in analysis of the coprimary endpoints.

We recruited 487 participants across 46 UK sites from Jan 6, 2014, to Sept 28, 2018 (appendix p 2–5), of whom 243 were randomly assigned to receive placebo and 244 to receive intravenous iron (figure 1). Six patients did not receive their intended randomised treatment (two assigned to placebo, four to intravenous iron). Eight patients withdrew consent (four in each group), of whom three withdrew between treatment and surgery, three between surgery and the 8-week visit, and two between the 8-week and 6-month visits. 23 patients did not undergo their planned surgery (13 placebo, ten intravenous iron), not including the three patients who had withdrawn consent before surgery. 46 patients had their surgery outside the prescribed timelines (26 placebo, 20 intravenous iron), and 20 patients did not have major open abdominal surgery (12 placebo, eight intravenous iron). For the coprimary endpoints, 474 (97%) patients were included in the intention-to-treat analysis and 388 (80%) patients were included in the per-protocol analysis.

The participants were well matched regarding baseline characteristics (table 1). The median age was 66 (IQR 54–72) years, and 267 (55%) were women. Most were American Society of Anesthesiologists grade II (61%) or III (26%). Comorbidities included hypertension (182 patients, 37·4%), diabetes (75 patients, 15%), previous myocardial infarction (32 patients, 7%), stroke or transient ischaemic attack (17 patients, 3%), renal disorders (76 patients, 16%), and respiratory problems (ie, breathlessness, chronic obstructive pulmonary disease, bronchitis, or asthma; 100 patients, 21%). Approximately half had never smoked and 41 (8%) of 487 patients were current smokers. Known iron deficiency and predisposing factors for iron deficiency were similar in the two groups.

**Table 1:** Baseline characteristics and surgical characteristics in PREVENTT

		**Placebo (n=243)**	**Intravenous iron (n=244)**
**Demographics**			
Age (years)		65 (50–72)	66 (57–72)
Men		101 (42%)	119 (49%)
Women		142 (58%)	125 (51%)
Ethnicity			
	White	217 (89%)	211 (86%)
	Afro-Caribbean	19 (8%)	14 (6%)
	Asian	6 (2%)	18 (7%)
	Other	1 (<1%)	1 (<1%)
**Haemoglobin (g/L)**			
<90		6 (2%)	7 (3%)
90–99		36 (15%)	35 (14%)
100–109		64 (26%)	55 (23%)
110–119		71 (29%)	80 (33%)
≥120		57 (23%)	61 (25%)
**Clinical measures**			
American Society of Anesthesiologists grade			
	I	31 (13%)	30 (12%)
	II	141 (58%)	147 (60%)
	III	65 (27%)	56 (23%)
	IV	1 (<1%)	1 (<1%)
	Missing	5 (2%)	10 (4%)
**Medical history**			
Acid reflux or stomach ulcer		54 (22%)	54 (22%)
Angina or chest pain		16 (7%)	15 (6%)
Bleeding tendencies		7 (3%)	11 (5%)
Breathlessness		28 (12%)	25 (10%)
Coeliac disease		2 (1%)	0
COPD, bronchitis, or asthma		37 (15%)	27 (11%)
Stroke or transient ischaemic attack		13 (5%)	4 (2%)
Diabetes		38 (16%)	37 (15%)
Inflammatory bowel disease		13 (5%)	13 (5%)
Iron deficiency		69 (28%)	70 (29%)
Heart failure		3 (1%)	9 (4%)
Hiatus hernia		23 (9%)	17 (7%)
Hypertension		93 (38%)	89 (36%)
Kidney or urinary problems		37 (15%)	39 (16%)
Liver disease		8 (3%)	14 (6%)
Myocardial infarction		20 (8%)	12 (5%)
Rheumatoid arthritis		12 (5%)	10 (4%)
Preoperative chemotherapy		59 (24%)	50 (20%)
Radiotherapy		6 (2%)	7 (3%)
**Smoking history**			
Never		116 (48%)	113 (46%)
Former		107 (44%)	108 (44%)
Current		19 (8%)	22 (9%)
Missing		1 (<1%)	1 (<1%)
**Current medication that affects bleeding**			
Aspirin		28 (12%)	23 (9%)
Clopidogrel		5 (2%)	3 (1%)
Other		25 (10%)	22 (9%)
Warfarin		4 (2%)	7 (3%)
**Iron tablets**			
Taking iron tablets		49 (20%)	46 (19%)
Missing		0	1 (<1%)
**Planned type of surgery**			
Complex major operation		85 (35%)	89 (36%)
Major		89 (37%)	87 (36%)
Major plus		69 (28%)	68 (28%)
**Surgical details**			
Surgery took place		228 (94%)	233 (95%)
**Time from treatment to surgery**			
Median (days)*		15 (12–22)	14 (12–21)
<10 days		5 (2%)	8 (3%)
>42 days		21 (9%)	12 (5%)
**Type of operation**			
Abdominal aortic aneurysm		4 (2%)	1 (<1%)
Colorectal		33 (14%)	38 (16%)
General		17 (7%)	21 (9%)
Gynaecological		75 (31%)	63 (26%)
Upper gastrointestinal		77 (32%)	81 (33%)
Urological		22 (9%)	29 (12%)
Anaesthetic time (min)		240 (161–320)	268 (180–376)
Surgery time (min)		145 (98–230)	179 (123–323)

**D**ata are n (%) or median (IQR). COPD=chronic obstructive pulmonary disease.

* Range 6–207 for placebo and 5–212 days for intravenous iron groups.

**Figure 2:**
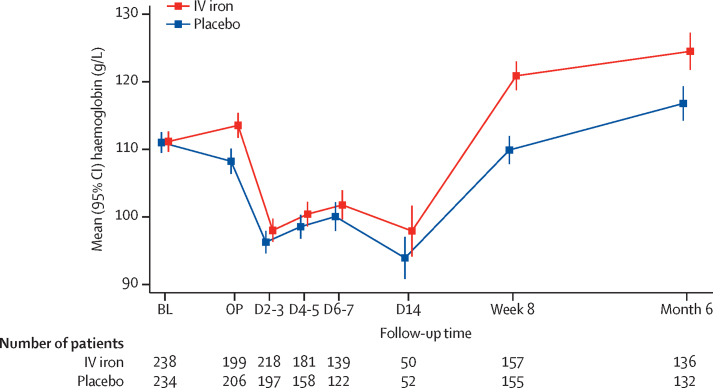
Mean haemoglobin concentrations of the trial participants by randomised treatment group Error bars show 95% CI. BL=baseline prerandomised treatment. OP=day of operation before surgery. D=day post operation (eg, D2–3=day 2 or 3 post operation). D2–3, D4–5, D6–7, and D14 measurements are only available for patients still hospitalised at that time. IV=intravenous.

Of the 487 patients randomly assigned, 461 (95%) underwent surgery—228 in the placebo group and 233 in the intravenous iron group. The median time from randomisation to surgery was 15 (IQR 12–22) days and was similar in the two groups. The groups were well balanced in terms of surgical complexity, with the most common operations being upper gastrointestinal (34%), gynaecological (30%), and colorectal (15%). At randomisation, haemoglobin concentrations were similar between the placebo (mean 111·0 [SD 11·9] g/L) and intravenous iron groups (mean 111·2 [11·8] g/L); this significantly increased in the intravenous iron group by the time of surgery (mean difference [MD] 4·7 g/L, 95% CI 2·7–6·8). Anaemia was corrected in 42 (21%) of 244 patients in the intravenous iron group compared with 21 (10%) of 243 patients in the placebo group (risk ratio 2·06, 95% CI 1·27–3·35). Haemoglobin concentrations (figure 2) were not significantly different in the immediate postoperative days but the intravenous iron group had significantly higher haemoglobin concentrations at 8 weeks (MD 10·7 g/L, 95% CI 7·8–13·7) and at 6 months following intervention (MD 7·3 g/L, 3·6–11·1).

A total of 474 (97%) of 487 patients (237 in each group) were included in the intention-to-treat analysis for the two coprimary endpoints. Overall, 136 patients (29%) received at least one blood transfusion or died between randomisation and 30 days after the index operation. There was no difference between the groups (67 placebo, 69 intravenous iron; risk ratio 1·03, 95% CI 0·78–1·37; p=0·84; absolute risk difference 0·8%, −7·3 to 9·0; table 2). 216 transfusion episodes occurred between randomisation and 30 days after the index surgery. There was no difference between the groups (111 placebo, 105 intravenous iron; rate ratio 0·98, 0·68–1·43; p=0·93; absolute rate difference 0·00, −0·14 to 0·15; table 2). Analysis of the coprimary endpoints at 6 months after the index operation also showed no difference between the groups (risk ratio for blood transfusion or death 0·99, 95% CI 0·77–1·28; rate ratio for transfusion episodes 0·92, 0·64–1·32).

**Table 2:** PREVENTT coprimary endpoints from randomisation to 30 days after operation

	**Placebo (n=243)**	**Intravenous iron (n=244)**	**Iron *vs* placebo (95% CI, p value)**
**Blood transfusion or death**			
Combined	67/237 (28%)	69/237 (29%)	1·03 (0·78–1·37, p=0·84)
Transfusion	67/237 (28%)	68/237 (29%)	..
Death	2/237 (1%)	2/237 (1%)	..
**Transfusion episodes**			
0	170/237 (72%)	169/237 (71%)	..
1	37/237 (16%)	49/237 (21%)	..
2	22/237 (9%)	9/237 (4%)	..
3	5/237 (2%)	5/237 (2%)	..
4	1/237 (<1%)	3/237 (1%)	..
5	1/237 (<1%)	1/237 (<1%)	..
6	1/237 (<1%)	1/237 (<1%)	..
Mean	0·47 (0·9)	0·44 (0·9)	0·98 (0·68–1·43, p=0·93)

Data are n/N (%), mean (SD), and risk or rate ratio (95% CI, p value). A transfusion episode is defined as receiving any volume of 1 unit (or part thereof) or more of packed red blood cells or any other blood product. Treatment effect is a risk ratio for the first coprimary endpoint (number of blood transfusions or deaths) and a rate ratio for the second coprimary endpoint; for the second coprimary endpoint, number of blood transfusions is the number of separate transfusions administered.

Analyses of the coprimary endpoints adjusting for age, sex, body-mass index, operation type, or haemoglobin concentration did not change the results (adjusted risk ratio for blood transfusion or death to 30 days 1·06, 95% CI 0·81–1·38; and adjusted rate ratio for transfusion episodes 0·99, 0·69–1·42; table 3). Treatment effect estimates in the per-protocol analyses were similar (risk ratio for blood transfusion or death to 30 days, 1·04, 95% CI 0·77–1·41; and rate ratio for transfusion episodes, 0·98, 95% CI 0·67–1·44).

**Table 3:** Prespecified subgroup analysis for coprimary endpoints

		**Placebo (n=243)**	**Intravenous iron (n=244)**	**Treatment effect (95% CI)**	**Interaction p value**
**Blood transfusion or death within 30 days**					
Age (years)					
	<70	44/156 (28%)	41/157 (26%)	0·93 (0·64–1·33)	..
	≥70	23/81 (28%)	28/80 (35%)	1·23 (0·78–1·95)	0·34
Central laboratory haemoglobin (g/L)					
	<100	23/42 (55%)	23/41 (56%)	1·02 (0·70–1·51)	..
	≥100	44/187 (24%)	45/190 (24%)	1·01 (0·70–1·45)	0·95
Sex					
	Female	42/139 (30%)	39/122 (32%)	1·06 (0·74–1·52)	..
	Male	25/98 (26%)	30/115 (26%)	1·02 (0·65–1·62)	0·91
Body-mass index (kg/m^2^)					
	<30	52/178 (29%)	51/161 (32%)	1·08 (0·79–1·50)	..
	≥30	15/57 (26%)	18/75 (24%)	0·91 (0·50–1·65)	0·62
Central laboratory ferritin (ng/mL)					
	<100	34/132 (26%)	34/128 (27%)	1·03 (0·69–1·55)	..
	≥100	32/98 (33%)	31/94 (33%)	1·01 (0·67–1·51)	0·94
Central laboratory TSAT (%)					
	<20	55/174 (32%)	49/163 (30%)	0·95 (0·69–1·31)	..
	≥20	8/50 (16%)	15/53 (28%)	1·77 (0·82–3·81)	0·13
Type of surgery					
	Complex major	25/83 (30%)	20/87 (23%)	0·76 (0·46–1·26)	..
	Major	17/86 (20%)	22/85 (26%)	1·31 (0·75–2·29)	..
	Major plus	25/68 (37%)	27/65 (42%)	1·13 (0·74–1·73)	0·32
**Blood transfusion episodes within 30 days**					
Age (years)					
	<70	0·5 (1·0)	0·4 (0·8)	0·79 (0·50–1·24)	..
	≥70	0·4 (0·7)	0·6 (1·1)	1·48 (0·79–2·77)	0·11
Central laboratory haemoglobin (g/L)					
	<100	0·8 (1·1)	0·8 (1·2)	1·07 (0·51–2·24)	..
	≥100	0·4 (0·9)	0·4 (0·8)	0·93 (0·61–1·41)	0·74
Sex					
	Female	0·5 (0·9)	0·4 (0·7)	0·92 (0·55–1·51)	..
	Male	0·4 (0·9)	0·5 (1·1)	1·07 (0·61–1·86)	0·69
Body-mass index (kg/m^2^)					
	<30	0·5 (0·9)	0·5 (1·0)	1·12 (0·73–1·72)	..
	≥30	0·5 (1·0)	0·3 (0·6)	0·68 (0·32–1·42)	0·25
Central laboratory ferritin (ng/mL)					
	<100	0·5 (1·0)	0·4 (0·9)	0·92 (0·55–1·52)	..
	≥100	0·5 (0·8)	0·5 (0·9)	1·07 (0·61–1·88)	0·70
Central laboratory TSAT (%)					
	<20	0·5 (1·0)	0·5 (1·0)	0·92 (0·60–1·41)	..
	≥20	0·3 (0·7)	0·4 (0·7)	1·55 (0·64–3·75)	0·29
Type of surgery					
	Complex major	0·6 (1·1)	0·4 (1·0)	0·77 (0·43–1·40)	..
	Major	0·3 (0·7)	0·3 (0·6)	1·24 (0·62–2·45)	..
	Major plus	0·6 (0·9)	0·6 (1·0)	1·09 (0·57–2·08)	0·56

Data are n/N (%), mean (SD), risk or rate ratio (95% CI), or p value. Treatment effect is risk ratio for risk of blood transfusion or death within 30 days (first coprimary endpoint) and rate ratio for blood transfusion episodes. TSAT=transferrin saturation.

Excluding large blood transfusions, 130 patients (64 placebo, 66 intravenous iron) were transfused with a total of 300 units of blood or blood products between randomisation and 30 days after operation. The mean transfusion rate at 30 days was 0·65 (SD 1·3) and 0·61 (1·3) in the placebo and intravenous iron groups, respectively (rate ratio 0·98, 95% CI 0·65–1·47; table 4).

**Table 4:** PREVENTT secondary and safety endpoints

		**Placebo (n=243)**	**Intravenous iron (n=244)**	**Iron *vs* placebo (95% CI)**
Units of blood*transfused from randomisation to 30 days after operation (excluding LBT)				
Mean		0·65 (1·3)	0·61 (1·3)	0·98 (0·65 to 1·47)^†^
Patients with ≥1 transfusion		64/237 (27%)	66/237 (28%)	..
Total units transfused		155	145	..
Units of blood*transfused from randomisation to 6 months after operation (excluding LBT)				
Mean		0·94 (2·0)	0·79 (1·6)	0·89 (0·60 to 1·32)^†^
Patients with ≥1 transfusion		73/224 (33%)	72/220 (33%)	..
Total units transfused		212	174	..
**Days alive and out of hospital within 30 days**				
Mean		19·8 (7·5)	19·7 (7·0)	−0·1 (−1·5 to 1·2)
**Postoperative complications**				
CD grade III or above to discharge		24/227 (11%)	22/233 (9%)	0·89 (0·52 to 1·55)^‡^
**MFI questionnaire**				
10-day assessment		50·5 (18·9)	53·2 (18·4)	1·2 (−1·1 to 3·4)
8-week assessment		53·9 (17·7)	52·9 (17·1)	−1·7 (−4·7 to 1·3)
6-month assessment		47·4 (19·1)	48·8 (18·9)	−0·1 (−3·5 to 3·2)
**EQ-5D-5L questionnaire**				
Utility score				
	10-day assessment	0·81 (0·21)	0·80 (0·20)	0·01 (−0·02 to 0·03)
	8-week assessment	0·77 (0·21)	0·79 (0·20)	0·02 (−0·01 to 0·05)
	6-month assessment	0·82 (0·21)	0·82 (0·22)	0·02 (−0·02 to 0·05)
Health score				
	10-day assessment	73·8 (19·6)	70·6 (20·5)	−0·8 (−3·5 to 1·9)
	8-week assessment	71·1 (19·5)	70·7 (19·4)	0·3 (−3·2 to 3·9)
	6-month assessment	76·2 (19·2)	75·0 (18·4)	0·2 (−3·4 to 3·8)
**ICU length of stay (days)**				
Median (IQR)		1 (0–3)	2 (0–3)	..
Range		0–23	0–33	..
**Hospital length of stay (days)**				
Median (IQR)		9 (5–14)	9 (7–14)	..
Range		1–46	1–118	..
**All-cause mortality**				
30 days		2/241 (1%)	2/239 (1%)	1·01 (0·14 to 7·10)^‡^
6 months		10/236 (4%)	12/238 (5%)	1·19 (0·52 to 2·70)^‡^
**Readmission to hospital for complications**				
Discharge to 8 weeks				
	Any readmission	51/234 (22%)	31/234 (13%)	0·61 (0·40 to 0·91)^‡^
	Total number of readmissions	71	38	0·54 (0·34 to 0·85)^†^
Discharge to 6 months				
	Any readmission	73/223 (32%)	58/227 (26%)	0·78 (0·58 to 1·04)^‡^
	Total number of readmissions	130	84	0·64 (0·44 to 0·92)^†^
**Safety outcomes to 6 months**§				
SAEs and SUSARs		23/240 (10%)	22/240 (9%)	0·96 (0·55 to 1·67)^‡^
Adverse reaction to trial therapy		5/240 (2%)	11/240 (5%)	2·20 (0·78 to 6·24)^‡^
Development of perioperative AKI		13/122 (11%)	11/137 (8%)	0·75 (0·35 to 1·62)^‡^

Data are mean (SD), n/N (%), n, median (IQR), range, or treatment effect (95% CI). LBT=large blood transfusion, defined as 4 or more units of blood transfused in a single transfusion episode (there were 9 LBTs in total). CD=Clavien–Dindo. ICU=intensive care unit. MFI=Multidimensional Fatigue Inventory. EQ-5D-5L=European Quality of Life: 5 Dimensions 5 Levels. SAE=serious adverse event. SUSAR=suspected unexpected serious adverse event. AKI=acute kidney injury.

* Total number of units of blood or blood products transfused. Treatment effect is either difference in mean or rate ratio (†) or risk ratio (‡).

§ Safety outcomes measured in the safety population.

At 6 months the overall blood transfusion rate was 29%, with 139 of 474 patients receiving at least one transfusion from randomisation until 6 months. Overall, packed red blood cells were transfused in 133 patients and only six patients received a different blood product in isolation; three had platelet and three had fresh frozen plasma transfusions. Otherwise, blood products were only used in combination with packed red cells in the setting of large transfusions. The mean transfusion rate was 0·94 (SD 2·0) and 0·79 (1·6) in the placebo and intravenous iron groups, respectively (rate ratio 0·89, 95% CI 0·60–1·32, p=0·56; table 4).

Postoperative complications were similar in the two groups, with 24 (11%) patients in the placebo group and 22 (9%) patients in the intravenous iron group experiencing significant (defined as Clavien–Dindo classification grade III or higher) postoperative complications (risk ratio 0·89, 95% CI 0·52–1·55). There was no difference in hospital stay or days alive and out of the hospital at 30 days (table 4). Similarly, there were no significant between-group differences for any of the HRQoL outcomes (table 4).

Readmissions to the hospital following surgery were significantly lower in the intravenous iron group in the first 8 weeks after the index operation (table 4). The number of patients readmitted for postoperative complications was 51 (22%) in the placebo group versus 31 (13%) in the intravenous iron group (risk ratio 0·61, 95% CI 0·40–0·91). Considering repeat readmissions, there were a total of 71 readmissions in the placebo group compared with 38 in the intravenous iron group (rate ratio 0·54, 95% CI 0·34–0·85). The most common reasons for readmission at 8 weeks were; general postoperative complications (36 [15%] of 234 patients in the placebo group and 25 [11%] of 234 patients in the intravenous iron group), general infections (seven [3%] in the placebo group and six [3%] in the intravenous iron group) and wound infections (eight [3%] in the placebo group and one [<1%] in the intravenous iron group). At 6 months, there were numerically fewer total readmissions in the intravenous iron group.

Mortality was similar in the two groups with ten (4%) deaths at 6 months in the placebo group and 12 (5%) in the intravenous iron group (risk ratio 1·19, 95% CI 0·52–2·70) and there were no significant differences between the two groups for any of the prespecified safety endpoints (table 4).

## Discussion

The use of intravenous iron in patients with anaemia before major open elective abdominal surgery increased haemoglobin concentrations before surgery but did not reduce the frequency of blood transfusion or mortality in the perioperative period relative to placebo. There was no reduction in the risk of postoperative in-hospital complications or length of hospital stay, and no benefits to quality of life. However, there was a reduced risk of readmission to hospital for complications in those patients who received intravenous iron.

PREVENTT reduces the uncertainty created by two previous small trials on the use of preoperative intravenous iron. The IVICA trial from Nottingham, UK, looked at 116 patients with anaemia undergoing colorectal cancer surgery and found that intravenous iron had no effect on blood transfusion use,^18^ whereas a smaller trial of 72 patients in Australia found that intravenous iron for patients with iron deficiency anaemia (ferritin <300 μg/L, transferrin saturation <25%) did reduce perioperative blood transfusion (12% *vs* 31%).^19^ PREVENTT suggests that preoperative intravenous iron has no significant effect on blood transfusion use in all patients with anaemia before major surgery.

Our findings are consistent with the existing evidence on iron therapy in non-cardiac patients. Trials of interventions to reverse anaemia, either with iron therapy or more liberal transfusion thresholds, have failed to show important clinical benefits,^20^, ^21^ despite observational evidence that anaemia is associated with poorer outcomes. This fact implies that treatments directed to the underlying causes of anaemia might be required to improve outcomes in this high-risk population.

The trial has several strengths, including allocation concealment, double-blinding, placebo control, high levels of adherence to the trial intervention (481/487), and low levels of attrition, with 474 of 487 participants providing data for the primary intention-to-treat analyses. There was no difference between the results of the per-protocol and intention-to-treat analyses or between the predefined subgroups, suggesting that non-adherence with other components of the protocol was unlikely to have influenced the trial result. The study included patients with a range of anaemia profiles including mild anaemia. These strengths, along with the broad inclusion criteria, clear documentation of process, and absence of effectiveness across a range of primary and secondary outcomes, support the validity and generalisability of the trial results.

One limitation was that preoperative iron deficiency was not specifically defined as an inclusion criterion although a predefined subgroup analysis was performed for those patients with a ferritin less than 100 ng/mL and transferrin saturations less than 20% in line with current guidelines for preoperative iron deficiency,^13^ of whom 57% had a ferritin less than 100 ng/mL and 76% had transferrin saturations less than 20% at inclusion and randomisation to the trial. There was no evidence of interaction between treatment in these predefined subgroups for the coprimary endpoints of the study.

The trial data suggest that there is no mortality or blood transfusion benefits to treating patients with a single 1000 mg dose of ferric carboxymaltose in the immediate preoperative period. Preoperative anaemia management is challenging in many health systems where preassessment is often carried out days before surgery, particularly for patients who have cancer for whom surgery is a priority, highlighted by recruitment to this trial where inclusion was a minimum of 10 days before surgery. The efficacy of intravenous iron was lower than expected with a minority of patients having their anaemia corrected before their date of surgery. The causal mechanism behind anaemia in the preoperative setting might require treatment with concurrent erythropoietin as seen in cardiac surgery.^22^ Erythropoietin is not licensed in such patients in the UK, but erythropoietin and intravenous iron has been recommended for anaemia in patients before orthopaedic surgery.^12^ Our findings have several important clinical implications. The treatment effect on mean haemoglobin values was higher after surgery than in the preoperative setting, despite no differences in type of surgery, bleeding, or transfusion volumes between the groups. The effect of preoperative intravenous iron and increased postoperative haemoglobin levels associated with reduced readmission to hospital for surgical complications merits further investigation. This effect might reflect an underlying mechanism of functional or absolute iron deficiency and anaemia of chronic disease with inflammation, and subsequent stimulus of blood loss at operation. Clinically, this finding raises the possibility that postoperative intravenous iron, before discharge from the hospital, might be effective at boosting haemoglobin levels in surgical patients during their recovery period. Postoperative intravenous iron would be easier and less expensive than intravenous iron preoperatively because the patient would already be in the hospital, being nursed and monitored in a hospital bed, and likely to have venous access in situ. This approach is unlikely, however, to be any more effective than preoperative intravenous iron in accruing benefits to the primary outcomes measured in our trial.

In conclusion, PREVENTT showed that intravenous iron was not superior to placebo when administered to patients with anaemia 10–42 days before elective major abdominal surgery with respect to reducing blood transfusion or death in the perioperative period.

## Data sharing

Research data and other material (eg, study protocol, statistical analysis plan, informed consent form) will be available to the scientific community, immediately on publication, with as few restrictions as possible. All requests should be submitted to the corresponding author for consideration. Researchers who provide a methodologically sound proposal likely to maximise the value of the data for patient and public benefit will be approved subject to review by a subgroup of the authors.
