# Supplementary material for: Preoperative intravenous iron to treat anaemia before major abdominal surgery (PREVENTT): a randomised, double-blind, controlled trial
Source: Lancet. 2020 Oct 24;396(10259):1353–61. doi: 10.1016/S0140-6736(20)31539-7 (PMC7581899; doi:10.1016/S0140-6736(20)31539-7)

THE LANCET

Supplementary appendix

This appendix formed part of the original submission and has been peer reviewed. We post it as supplied by the authors.

Supplement to: Richards T, Rao Baikady R, Clevenger B, Butcher A, Abeysiri S, Chau M, et al. Preoperative intravenous iron to treat anaemia before major abdominal surgery (PREVENTT): a randomised, double-blind, controlled trial. *Lancet* 2020; published online Sept 4. http://dx.doi.org/10.1016/S0140-6736(20)31539-7.

Supplementary material

Trial Steering Committee
Andrew Bradbury (Chair), Toby Richards (Chief Investigator), Trevor Burley, Shelley Van Loen, Stefan Anker, Andrew Klein, Iain Macdougall, Gavin Murphy, Martin Besser and Isabel Unsworth.
Observers - Tim Clayton, Tim Collier, Kimberley Potter, Sandy Abeysiri, Richard Evans, Rosemary Knight, Rebecca Swinson, Laura Van Dyck and Jane Keidan.

Data Safety and Monitoring Committee
Lorna Williamson (Chair), Angela Crook, and John Pepper
Unblinded statisticians supporting the DSMC were Joanna Dobson, Simon Newsome, Tom Godec and Matthew Dodd.

Project Management Group
Toby Richards, Laura Van Dyck, Richard Evans, Sandy Abeysiri, Ben Clevenger, Anna Butcher, Rebecca Swinson, Tim Collier and Kimberley Potter.

Study Design and Support
Stefan Anker, John Kelly, Steven Morris, John Browne, Jane Keidan, Michael Grocott, Marisa Chau and Rosemary Knight.

Trial Statistician
Timothy Collier

Randomisation Service
Sealed Envelope™

Blood Sample Analysis
The Doctors Laboratory

Contributing Sites & Investigators

Royal Marsden NHS Foundation Trust *(118 patients, opened April 2, 2014)*
Ravishankar Rao Baikady (PI), Ethel Black, Helen Lawrence, Maria Kouthra, Katherine Horner, Sham Jhanji, Ed Todman, Zoe Keon-Cohen, Martin Rooms, Judith Tomlinson, Ian Bailes, Susanna Walker, Katrina Pirie, Michelle Gerstman, Ramanathan Kasivisvanathan, Sophie Uren, David Magee, Alex Eeles, Rob Anker, Jamie McCanny, Michelle O'Mahony, Toby Reynolds, Sian Batley, Aoife Hegarty, Simon Trundle, Francesca Mazzola, Kate Tatham, Alina Balint, Ben Morrison, Matthew Evans, Ching Ling Pang, Lorna Smith, Charlotte Wilson, Victoria Sjorin, Poonam Khatri, Marco Wilson, David Parkinson

University College London Hospitals NHS Foundation Trust *(38 patients, opened September 1, 2013)*
James Crosbie (PI), Khaled Dawas (PI), Deborah Smyth, Georgia Bercades, Jung Ryu, Anna Reyes, Gladys Martir, Laura Gallego, Alison Macklin, Magda Rocha, Dr Karen Tam, Dr David Brealey

Guy's and St Thomas' NHS Foundation Trust *(25 patients, opened May 7, 2014)*
Jugdeep Dhesi (PI), Catriona Morrison, Joanna Hardwick, Jude Partridge, Philip Braude, Andrew Rogerson, Nymah Jahangir, Clare Thomson, Lizzie Biswell, Jason Cross, Ffion Pritchard, Aminata Mohammed, Deirdre Wallace, Ma Gem Galat, Jane Okello, Rebecca Symes, Mollika, Chakravorty, Josette Leon, Angela Cape (pharmacy), Charlotte Gibbs (pharmacy)

Sheffield Teaching Hospitals NHS Foundation Trust *(24 patients, opened November 11, 2013)*
Sumayer Sanghera (PI), Andy Dennis, Faith Kibutu, Joyce Fofie, Sarah Bird, Abiola Alli, Yvonne Jackson

North Bristol NHS Trust *(23 patients, opened February 4, 2014)*
Salah Albuheissi (PI), Carol Brain, Connie Shiridzinomwa

Royal Cornwall Hospitals NHS Trust *(23 patients, opened September 19, 2013)*
Catherine Ralph (PI), Belinda Wroath, Fiona Hammonds, Benita Adams, John Faulds, Sara Staddon

King's College Hospital NHS Foundation Trust *(18 patients, opened February 26, 2016)*
Timothy Hughes (PI), Sian Saha, Clare Finney, Clair Harris, Clare Mellis, Lucy Johnson, Paul Riozzi, Adam Yarnold, Fraser Buchanan, Philip Hopkins, Louise Greig, Harriet Noble

University Hospital Southampton NHS Foundation Trust *(18 patients, opened January 7, 2014)*
Mark Edwards (PI), Mike Grocott, James Plumb, David Harvie, Ahilanandan Dushianthan, Mai Wakatsuki, Samantha Leggett, Karen Salmon, Clare Bolger, Rachel Burnish, James Otto, Gurinder Rayat, Kim Golder, Pauline Bartlett, Sitara Bali, Leanne Seaward, Beverley Wadams, Bryony Tyrell, Hannah Collins, Natasha Tantony, Rosie Geale, Amber Wilson, Darran Ball

Oxford University Hospitals NHS Foundation Trust *(15 patients, opened October 22, 2015)*
Ian Lindsey (PI), Debbie Barker, Madeleine Thyseen

South Tees Hospitals NHS Foundation Trust *(11 patients, November, 20, 2014)*

Patrick Chiam (PI), Carol Hannaway, Kerry Colling

The Hillingdon Hospitals NHS Foundation Trust *(11 patients, opened October 4, 2013)*
Cheryl Messer (PI), Neil Verma, Mariam Nasseri, Gail Poonawala, Abbie Sellars, James Harris (PI), Pratyuja Mainali

Mid Essex Hospital Services NHS Trust *(10 patients, opened March 11, 2015)*
Toby Hammond (PI), Al Hughes, David O'Hara, Fiona McNeela, Lauren Shillito

Leeds Teaching Hospitals NHS Trust *(10 patients, opened November 12, 2014)*
Alwyn Kotze (PI), Catherine Moriarty

York Teaching Hospital NHS Foundation Trust *(10 patients, opened December 17, 2013)*
Jonathan Wilson (PI), Simon Davies, David Yates, Joe Carter, Jon Redman, Sara Ma, Kate Howard, Heidi Redfearn, Danielle Wilcock

Imperial College Healthcare NHS Trust *(9 patients, opened November 6, 2015)*
Justine Lowe (PI), Asela Dharmadasa (PI), Tamara Alexander, Jasmine Jose, Gillian Hornzee, Fatima Akbar, Severine Rey, Anoop Patel, Samantha Coulson, Rajan Saini, Joseph Santipillai, Thomas McCretton, Ian Bailes (PI), Jamie McCanny, Kiran Chima, Karen Collins, Byiravey Pathmanathan, Anjalee Chattersingh, Laura McLeavy, Zayneb Al-Saadi

Salford Royal NHS Foundation Trust *(9 patients, opened November 17, 2014)*
Manju Patel (PI), Sofia Skampardoni, Rajkumar Chinnadurai, Vicky Thomas, Anne Keen, Katherine Pagett, Clare Keatley, Jason Howard, Leigh Willoughby (PI), Marie Greenhalgh

The Dudley Group NHS Foundation Trust *(8 patients, opened March 8, 2016)*
Stephen Jenkins (PI), Ranjit Gidda, Angela Watts

Royal Liverpool and Broadgreen University Hospitals NHS Trust *(8 patients, opened February 20, 2015)*
Chris Breaton (PI), Jane Parker

Royal Free London NHS Foundation Trust *(8 patients, opened December 19, 2013)*
Susan Mallett (PI), Sarah James

Wye Valley NHS Trust *(7 patients, opened May 20, 2015)*
Lisa Penny (PI), Kim Chan, Tamsin Reeves, Marisa Catterall, Sue Williams, Janine Birch, Kate Hammerton, Nicola Williamson, Anni Thomas, Melanie Evans, Lily Mercer, Gill Horsfield, Claire Hughes

Blackpool Teaching Hospitals NHS Foundation Trust *(7 patients, opened September 1, 2014)*
Jason Cupitt (PI), Emma Stoddard

Liverpool Women's NHS Foundation Trust *(6 patients, May 4, 2018)*
Helen McNamara (PI), Chloe Birt

North West Anglia NHS Foundation Trust (Peterborough) *(6 patients, opened December 12, 2015)*
Alexander Hardy (PI), Robert Dennis, Deborah Butcher, Susie O'Sullivan, Alan Pope

University Hospital of South Manchester NHS Foundation Trust *(6 patients, opened September 29,2014)*
Sumaya Elhanash (PI), Stephen Preston, Helen Officer, Andrea Stoker, Stuart Moss, Alison Walker, Anna Gipson, Julie Melville, Joanne Bradley-Potts, Richard McCormac, Vivienne Benson, Kirsty Melia, Julie Fielding, Wendy Guest

Swansea Bay University Health Board *(6 patients, opened July 8, 2014)*
Simon Ford (PI)

Gloucestershire Hospitals NHS Foundation Trust *(5 patients, opened February 22, 2018)*
Henry Murdoch (PI), Susan Beames, Paula Townshend, Kayleigh Collins, Jon Glass, Bethan Cartwright

Aintree University Hospital NHS Foundation Trust *(5 patients, opened March 11, 2016)*
Balsam Altemimi (PI), Lucy Berresford

Royal Surrey County Hospital NHS Foundation Trust *(5 patients, opened September 19, 2014)*
Chris Jones (PI), Leigh Kelliher, Sam de Silva, Katie Blightman

Central Manchester University Hospitals NHS Foundation Trust *(5 patients, opened Juner 27, 2014)*
Kate Pendry (PI), Lebina Pinto

Barts Health NHS Trust *(5 patients, opened May 9, 2014)*
Shubha Allard (PI), Louise Taylor

The Newcastle upon Tyne Hospitals NHS Foundation Trust *(5 patients, opened March 7, 2014)*
Ahmed Chishti (PI), Julia Scott

Norfolk and Norwich University Hospitals NHS Foundation Trust *(4 patients, opened November 6, 2015)*
Debbie O'Hare (PI), Michael Lewis

Pennine Acute Hospitals NHS Trust *(4 patients, July 22, 2015)*
Zahid Hussain (PI), Karen Hallett, Susan Dermody, Carolyn Corbett, Louise Morby

Dorset County Hospital NHS Foundation Trust *(4 patients, opened December 6, 2013)*
Matthew Hough (PI), Sarah Williams, Patricia Williams, Sarah Horton, Pauline Ashcroft, Anthony Homer

Royal Edinburgh Hospital - NHS Lothian *(3 patients, opened March 4, 2016)*
Alastair Lang, Heidi Dawson, Ewen Harrison (PI)

Royal Devon and Exeter NHS Foundation Trust *(3 patients, opened May 12, 2014)*
John Thompson (PI)

North West Anglia NHS Foundation Trust (Hinchingbrook) *(1 patient, opened February 1,2018)*
Vimal Hariharan (PI), Vanessa Goss

Basildon and Thurrock University Hospitals NHS Foundation Trust *(1 patient, opened October 4, 2017)*
Ramachandran Ravi (PI), Georgina Butt, Mark Vertue

Nottingham University Hospitals NHS Trust *(1 patient, opened May 27, 2016)*
Austin Acheson (PI), Oliver Ng, Debbie Bush, Edward Dickson, Amy Ward

Brighton and Sussex University Hospitals NHS Trust *(1 patient, opened March 7, 2014)*
Sophie Morris (PI), R&D Management

Maidstone and Tunbridge Wells NHS Trust *(1 patient, opened October 10, 2013)*
Andrew Taylor (PI), Rebecca Casey

Countess of Chester Hospital NHS Foundation Trust *(0 patients, opened April 26, 2017)*
Lawrence Wilson (PI), Dale Vimalachandran, Maria Faulkner, Helen Jeffrey, Claire Gabrielle, Sharon Martin

Gateshead Health NHS Foundation Trust *(0 patients, opened November, 232015)*
Andrew Bracewell (PI), Jenny Ritzema, David Sproates

Northampton General Hospital NHS Trust *(0 patients, opened December 22, 2014)*
Farhad Alexander-Sefre (PI)

West Suffolk NHS Foundation Trust *(0 patients, opened September 16, 2014)*
Christiane Kubitzek (PI), Sally Humphreys, James Curtis, Paul Oats, Sandra Swann, Abbie Holden, Claire Adam

University Hospitals Bristol NHS Foundation Trust *(0 patients, opened May 19, 2014)*
Louise Flintoff, Claudia Paoloni (PI), Karen Bobruk

Inclusion criteria

Patients who met the following criteria at the start of treatment were eligible for the study:

1. At least 18 years of age and signed written informed consent.
2. Undergoing elective major open abdominal surgery.
3. Benign or malignant disease.
4. Major surgery was defined as an operation of anticipated duration of more than one hour.
5. Screening haemoglobin (Hb) greater than or equal to 90 g/L (9.0 g/dL) but below or equal to 120 g/L (12.0 g/dL) in women or 130 g/L (13.0 g/dL) in men within four weeks of randomisation.
6. Randomisation and administration of study infusion a minimum of 10 days and maximum 42 days before planned operation.
7. Negative pregnancy test for women of childbearing potential (within last 7 days), and agreed to use effective form of contraception until 6 weeks post treatment.
8. Laboratory data used for determination of eligibility at the baseline visit not older than four weeks.

Exclusion criteria

Patients who, at the start of treatment, met any of the following criteria were not eligible for the study:

1. Undergoing laparoscopic surgery.
2. Body weight under 50 kg.
3. Known history of acquired iron overload, or family history of haemochromatosis or thalassemia or TSAT >50%.
4. Known reason for anemia (e.g., untreated B12 or folate deficiency or haemoglobinopathy).
5. Known hypersensitivity to ferric carboxymaltose (Ferinject®) or its excipients.
6. Temperature >37.5°C or patient on non-prophylactic antibiotics.
7. Known chronic liver disease.
8. If clinically indicated for the patient to have LFTs as part of pre-assessment for surgery and this screening alanine transaminase (ALT) or aspartate transaminase (AST) is above three times the upper limit of the normal range.
9. Received erythropoietin or intravenous iron therapy in the previous 12 weeks.
10. Immunosuppressive therapy (for organ transplantation) or renal dialysis (current or planned within the next 12 months).
11. Severe asthma or severe allergy (requiring hospitalisation within the last 12 months).
12. Unfit for elective surgery.
13. Pregnancy or lactation.
14. Inability to fully comprehend and/or perform study procedures in the investigator’s opinion.
15. Patient involvement in another IMP trial within the previous 4 weeks, prior to randomisation.
16. Involvement in another IMP trial, following randomisation, that may impact on the results of the PREVENTT trial.

Statistical Methods: Secondary Outcome Analyses

Total number of units of blood or blood components transfused between randomization to (i) 30 days and (ii) 6 months post index operation, excluding large blood transfusions.

The mean (SD) and median (IQR) number of units of blood were reported by treatment group. The total number of units transfused were also categorised (0,1,2,3,4,5,6+) and the frequency and percentage of patients in each group reported. Rate ratios (treatment versus placebo) and 95% confidence intervals were calculated using a negative binomial regression model. A p-value was calculated using a likelihood-ratio test.

Days alive and out of hospital (DAOH) until 30 days post index operation

The number of DAOH from the date of the index operation until 30 days post index operation was calculated for each patient using the method described by Ariti et al. Discharge to a nursing home or other care facility was not considered as being out of hospital. The number and percentage of patients lost to follow-up before 30 days post index operation was reported for each treatment group; these patients were excluded from this analysis. The mean (SD) and median (IQR) DAOH was reported by treatment group. DAOH at 30 days post index event was analysed with a linear regression model. A sensitivity analysis was also carried out comparing DAOH between the treatment groups using a Wilcoxon Rank Sum Test.

Postoperative complications from index operation to date of discharge

Postoperative complications during the inpatient period were classified using the Clavien-Dindo (CD) system. For each patient the most severe postoperative complication was identified. The number and proportion of patients with any moderate or severe complication (CD Grade III or above) was reported by treatment group. A risk ratio (treatment versus placebo), 95% CI and p-value were calculated. Additionally, the worst postoperative complication for each patient was categorised as none/mild (CD I or II), CD III, CD IV and CD V and the number and percentage of patients in each category was reported by treatment group. Between group comparisons were made using a test for trend.

Quality of life

The secondary outcomes included health-related quality of life as measured by the EuroQoL instrument (EQ-5D-5L), fatigue as measured by the Multidimensional Fatigue Inventory (MFI) and the patient's overall perception of outcome as measured by a Single Question Outcome Measure (SQOM). The EQ-5D-5L produces two scores: a 'descriptive index' that can range from -0.59 to 1.00 and a visual-analogue measure of overall health that can range from 0-100. Higher scores on the EQ-5D-5L represent better outcome. The MFI is a 20-item measure that produces domain-specific scores for general fatigue, physical fatigue, reduced activity, reduced motivation and mental fatigue which can range from 4 to 20. A total MFI score that can range from 20 to 100 can also be calculated. Higher MFI scores represent greater fatigue. The SQOM asked the patient to record how their condition had changed since starting their participation in the study. Patients could choose from seven response options ranging from 'has much improved' to 'is much worse'. Scores could range from -3 to +3, with higher scores representing greater improvement.

Continuous outcome measures at each follow-up time point for the two trial groups were compared using multivariate linear regression with adjustment for covariates including baseline levels of the relevant outcome measure.

Change in hemoglobin levels from randomisation to (i) Day of operation (prior to surgery), (ii) 8 weeks and (iii) 6 months post index operation

Mean (SD) hemoglobin (g/dl) at randomisation and at each visit was reported by treatment group. Mean (SD) change in hemoglobin (g/dl) was reported at each visit by treatment group. Differences between treatment groups in mean change in hemoglobin levels from randomization to the day of index operation were analysed using analysis of covariance (ANCOVA) with baseline hemoglobin level included in the model. It may be necessary to examine haemoglobin for transformations to adhere to the assumptions of the ANCOVA model. The adjusted difference in mean change (treatment versus placebo) in hemoglobin was reported along with a 95% CI and p-value from an F-test.

Correction of anemia at day of index operation

The number and percentage of patients with anemia corrected (males Hb>13 g/dL and female Hb>12 g/dL) at the day of the index operation was reported by treatment group. A risk ratio (treatment versus placebo) and 95% CI for the correction of anemia at the day of the index operation were estimated using binomial regression (binary outcome with a log link). A p-value was calculated using a likelihood-ratio test. Patients whose operations were cancelled were excluded from this analysis unless they had a Hb measurement on the planned day of surgery. The number and percentage of patients missing was reported by treatment group.

Risk of blood transfusion or death, excluding large blood transfusion, from randomization to (i) 30 days and (ii) 6 months post index operation
The same statistical methods as described for the first co-primary endpoint were used for these analyses.

ICU and total hospital length of stay
ICU and total length of hospital stay relating to the index operation was summarised using median, inter-quartile range and the range for each treatment group. Differences between groups were tested using the Mann-Whitney U test.

Readmission to hospital at (i) 8 weeks and (ii) 6 months post index operation
The number and percentage of patients readmitted to hospital was reported by treatment group at each of the follow-up visits, and reasons for readmission reported. The same statistical methods were used as described above for the first co-primary endpoint. Kaplan-Meier curves were plotted to visually compare readmission to hospital between treatment groups at 8 weeks and 6 months. For these analyses, analysis time started from the date of discharge from the index operation. For any patients that did not have the index operation, the planned day of operation plus the median length of hospital stay in that treatment arm were used as the start of analysis time. A Cox proportional hazards model was used to obtain a hazard ratio (and 95% confidence interval) comparing readmissions in treatment groups at 8 weeks and 6 months. P-values were calculated using a log-rank test.

All-cause mortality at (i) 8 weeks and (ii) 6 months post index operation
Risk ratios (treatment versus placebo) and 95% CI for all-cause mortality at 8 weeks and 6 months post index operation were estimated using binomial regression (binary outcome with a log link). A p-value was calculated using an LRT.
Kaplan-Meier curves were plotted to visually compare mortality between treatment groups from randomization to 8 weeks and 6 months post-operatively. A Cox proportional hazards model was used to obtain a hazard ratio (and 95% confidence interval) comparing mortality in treatment groups at 8 weeks and 6 months.

Safety and other related efficacy outcomes
Large blood transfusion.
Any reaction or side effect from trial therapy.
Any reaction or side effect from whole blood or blood product (transfusion reaction).
Serious Adverse Events (SAEs) and Suspected Unexpected Serious Adverse Events (SUSARs).
Development of perioperative AKI.
Concomitant medications.
For each of the above safety outcomes, the number and percentage of patients were reported by treatment group for each outcome. Risk ratios and 95% CIs were calculated, and p-values calculated using a chi-squared test or Fisher's exact test as appropriate.

Recruitment by Site and by Month

Recruitment by site

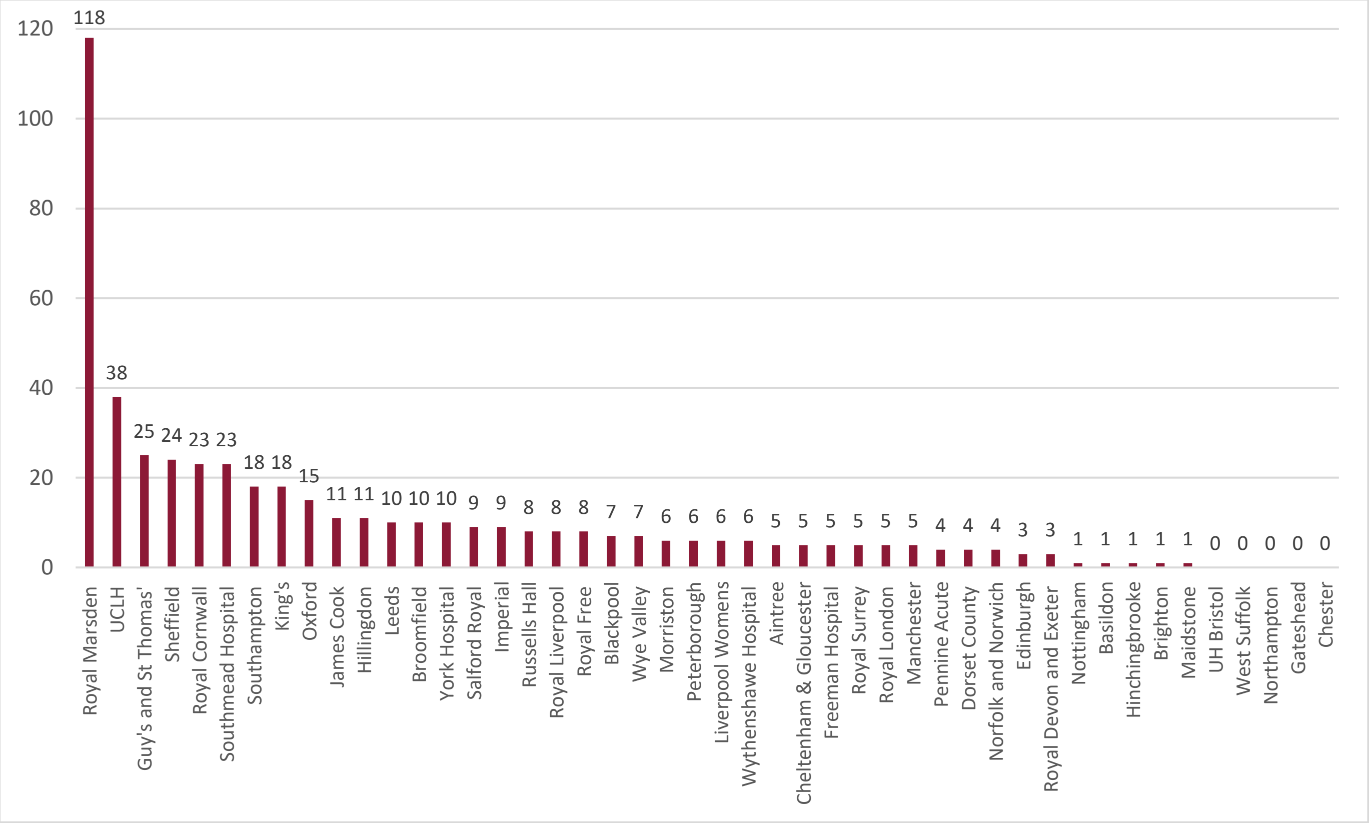

Recruitment by month

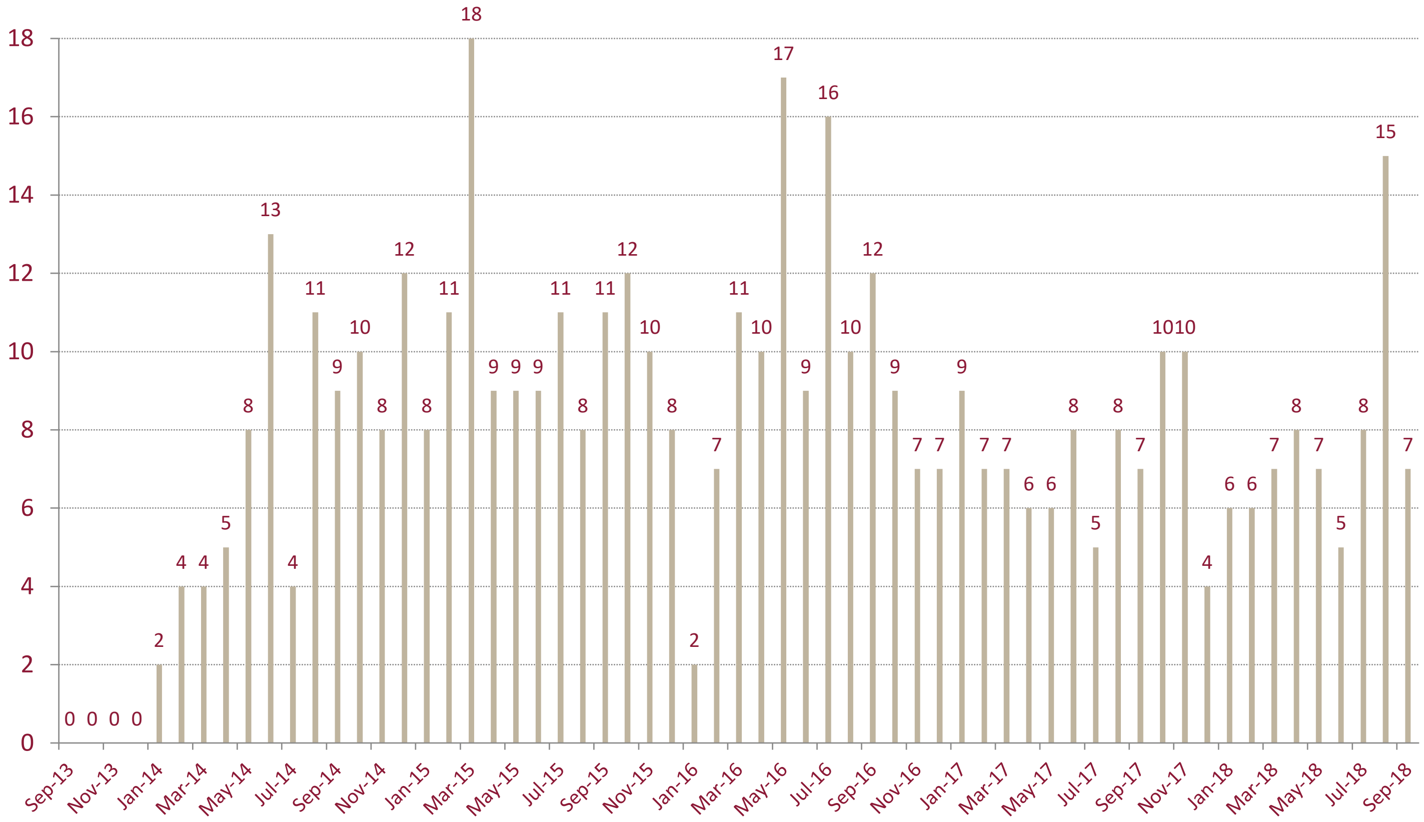

Cumulative recruitment

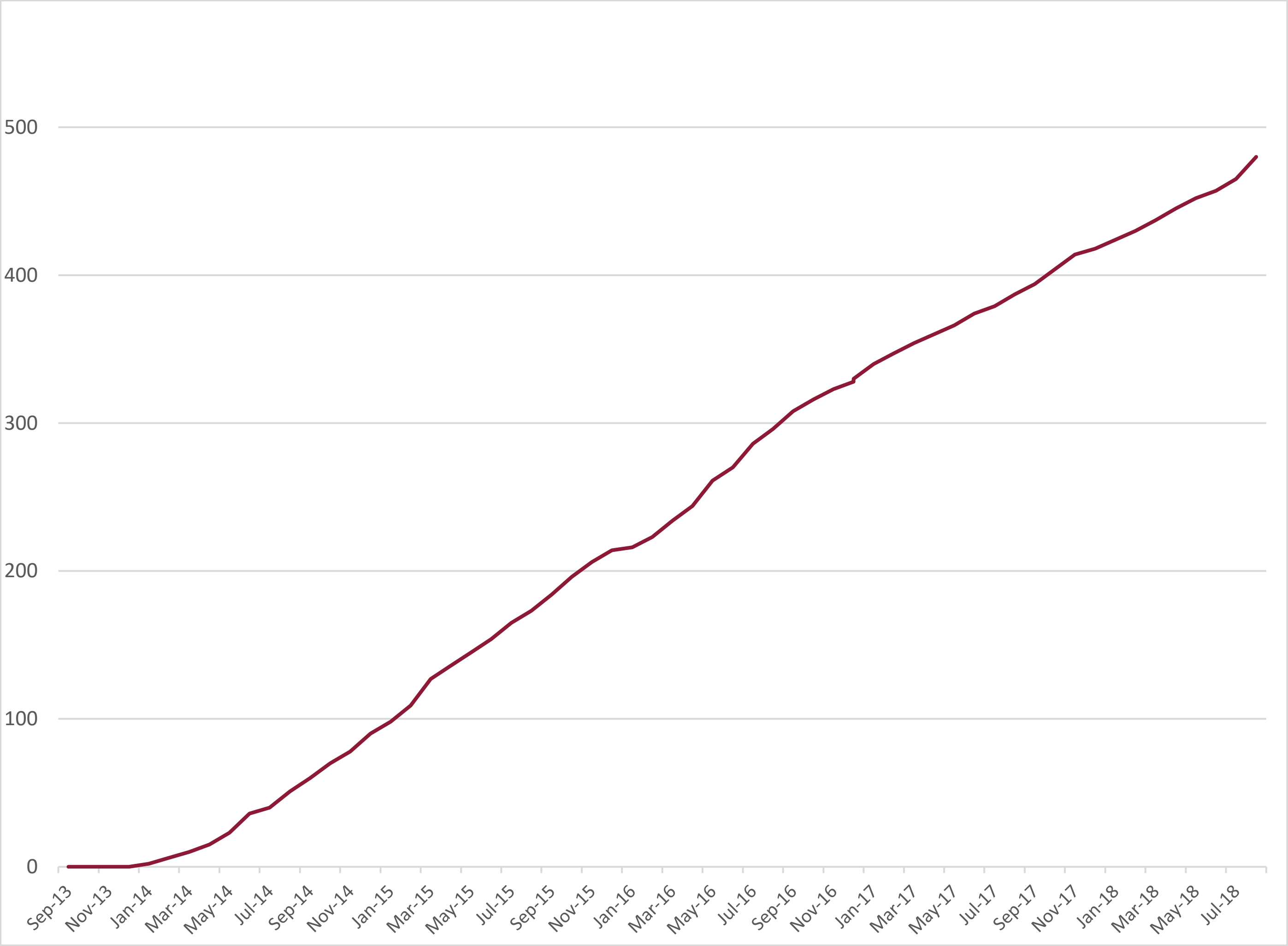

Supplement: Supplementary appendix [file mmc1.pdf]
